# Broadening the horizon: potential applications of CAR-T cells beyond current indications

**DOI:** 10.3389/fimmu.2023.1285406

**Published:** 2023-11-27

**Authors:** Hendrik Karsten, Ludwig Matrisch, Sophia Cichutek, Walter Fiedler, Winfried Alsdorf, Andreas Block

**Affiliations:** ^1^ Faculty of Medicine, University of Hamburg, Hamburg, Germany; ^2^ Department of Rheumatology and Clinical Immunology, University Medical Center Schleswig-Holstein, Lübeck, Germany; ^3^ Faculty of Medicine, University of Lübeck, Lübeck, Germany; ^4^ Department of Oncology, Hematology and Bone Marrow Transplantation with Division of Pneumology, University Medical Center Eppendorf, Hamburg, Germany

**Keywords:** CAR-T-cell therapy, T-cell malignancies, AML, CML, CLL, lymphoma, hairy cell leukemia, Waldenström’s macroglobulinemia

## Abstract

Engineering immune cells to treat hematological malignancies has been a major focus of research since the first resounding successes of CAR-T-cell therapies in B-ALL. Several diseases can now be treated in highly therapy-refractory or relapsed conditions. Currently, a number of CD19- or BCMA-specific CAR-T-cell therapies are approved for acute lymphoblastic leukemia (ALL), diffuse large B-cell lymphoma (DLBCL), mantle cell lymphoma (MCL), multiple myeloma (MM), and follicular lymphoma (FL). The implementation of these therapies has significantly improved patient outcome and survival even in cases with previously very poor prognosis. In this comprehensive review, we present the current state of research, recent innovations, and the applications of CAR-T-cell therapy in a selected group of hematologic malignancies. We focus on B- and T-cell malignancies, including the entities of cutaneous and peripheral T-cell lymphoma (T-ALL, PTCL, CTCL), acute myeloid leukemia (AML), chronic myeloid leukemia (CML), chronic lymphocytic leukemia (CLL), classical Hodgkin-Lymphoma (HL), Burkitt-Lymphoma (BL), hairy cell leukemia (HCL), and Waldenström’s macroglobulinemia (WM). While these diseases are highly heterogenous, we highlight several similarly used approaches (combination with established therapeutics, target depletion on healthy cells), targets used in multiple diseases (CD30, CD38, TRBC1/2), and unique features that require individualized approaches. Furthermore, we focus on current limitations of CAR-T-cell therapy in individual diseases and entities such as immunocompromising tumor microenvironment (TME), risk of on-target-off-tumor effects, and differences in the occurrence of adverse events. Finally, we present an outlook into novel innovations in CAR-T-cell engineering like the use of artificial intelligence and the future role of CAR-T cells in therapy regimens in everyday clinical practice.

## 1 Introduction

The diverse cluster of mature hematologic malignancies can broadly be classified into five groups using genetic, molecular, and clinical parameters: mature B-cell neoplasms, classic Hodgkin-Lymphomas, mature T- and natural killer (NK)-cell-neoplasms, histiocytic and dendritic cell neoplasms, and immunodeficiency-associated lymphoproliferative disorders (1).

These groups feature a wide spectrum of pathophysiological mechanisms of disease, pathological features, and therapeutic options. The clinical characteristics of these diseases differ significantly: While some, like CLL, do not necessarily require therapy upon diagnosis (2), diseases such as aggressive T-cell malignancies still present a highly complex clinical challenge with a median overall survival of less than 12 months (3). Patient groups like people living with HIV (PLWH) are at increased risk of developing hematologic malignancies (4). Some diseases are more common in pediatric (acute leukemias) or geriatric (CLL) patients (5). The treatment and management of these malignancies have significantly benefited from the implementation of CAR-T-cell therapies and this technology remains the most successful of several cellular immunotherapies developed in the 21^st^ century (6, 7).

Research currently focuses on expanding this technology towards treatment of solid tumors. Other possible areas of application are infectious diseases (e.g. HIV, HBV, other viral and fungal infections), and auto-immune disorders (such as rheumatoid arthritis, systemic lupus erythematosus). While these areas are prominently featured in the current CAR-T-cell research landscape, there are also approaches for its implementation in novel hematologic malignancies. Currently, many of these diseases are treated utilizing a combination of chemotherapy and immunotherapy, and in some cases, autologous or allogeneic hematopoietic stem cell transplantation (allo-HSCT), leading to high response rates and potential long-lasting complete remission. However, patients with relapsed or refractory disease often have a poor prognosis (8, 9). Furthermore, particular patient populations like heavily pretreated patients and those over the age of 60 years often suffer from reduced response to therapy or limited life expectancy (10).

To extend the remarkable achievements of CAR-T-cell therapy to more diseases and patient populations, researchers are currently employing an array of different technologies and approaches. These include CRISPR/Cas9- and TALEN-based (Transcription activator-like effector nucleases) gene-editing, OMICs methods (including transcriptomics, surfaceomics, and proteomics), nanotechnology, single-cell technologies, and advanced combination regimens with established therapies. Through these approaches, more and more malignant entities become possible targets of CAR-T-cell therapy (11–13).

However, severe adverse events including immune effector cell-associated neurotoxicity syndrome (ICANS) and cytokine release syndrome (CRS) (14) as well as T-cell dysfunction, resistance, and tumor escape mechanisms still pose difficulties (15, 16). Novel approaches for the management of these adverse events are currently under development, including subcutaneous injection of IL-6-adsorbing hydrogel (17) and the implementation of inducible “on-and-off” systems (18). Recent research has highlighted the possibility to employ sequential CAR-T-cell therapies to tackle post-CAR-T-cell relapse, a major cause of death in CAR-T-cell treated patients (19). Additionally, tools are being developed to aid in the management of CAR-T-cell patients and improve clinical care (20).

After initial hopes that CAR-T cells could present a potentially universal therapeutic approach for malignant diseases, researchers are currently understanding that CAR-T cells are most likely just one of several options in personalized therapies (21). Therefore, other innovative immunotherapeutic strategies are being investigated, including different immune cell groups, oncolytic viruses, technologies like T-cell engagers, T-cell receptor (TCR) engineering, and combinations of these technologies with CAR-T cells (22). The ultimate goal of these approaches and innovations is the improvement of clinical outcomes like overall survival (OS), progression-free survival (PFS), prevention of adverse events, improvements in patients’ quality of life, and above all the identification of curative therapeutic strategies.

While many of these techniques are still in their early development, several CAR-T-cell therapies have already been approved and are utilized in the clinical setting. Therefore, we highlight potential applications of CAR-T-cell technology in hematologic malignancies beyond the currently approved indications. We also provide an overview of the current research and possible innovations regarding different lymphomas and leukemias. We further focus on aspects such as safety, efficacy, and organizational issues.

## 2 T-cell malignancies

### 2.1 Current state of treatment

T-cell malignancies include lymphomas and leukemias originating from T cells and their precursor cells. Within the past decade, the prognosis of acute lymphatic T-cell leukemia (T-ALL) has improved, especially due to new chemotherapy protocols and monitoring of minimal residual disease (MRD) after therapy (23). However, these diseases still pose a difficult task for oncologists as the treatment and management of complications remain complex with poor outcome (24). Especially patients with cases of refractory or relapsing disease rarely respond well to established salvage protocols (25). Standard therapy regimens include chemotherapy, histone deacetylase inhibitors (HDACi) and monoclonal antibodies targeting antigens such as CD30 or CCR4 (26).

The lack of malignant T-cell-specific target antigens for CAR-T cells is one of the main difficulties as most of the targeted antigens in T-cell malignancies (such as CD3, CD5, CD7) are expressed by healthy T cells as well. A T-cell depleting therapy would lead to a complete eradication of T cells, resulting in detrimental infectious complications (27). The second fundamental challenge in employing CAR-T-cell therapy for T-cell malignancies is the apheresis of exclusively healthy T cells from the patient in order to generate CAR-T cells without contamination with circulating tumor cells (28).

### 2.2 CD7-directed CAR-T-cell therapy

To account for these limitations, a CAR-T-cell therapy needs to be able to distinguish between healthy and malignant T cells to a required extent. This poses a substantial challenge to researchers as the expressed proteins on healthy and malignant T cells differ only marginally (29). The most comprehensively studied T-cell-specific target is CD7 due to its abundant expression in T-cell malignancies compared to limited presence on healthy T cells (30).

**Figure 1:**
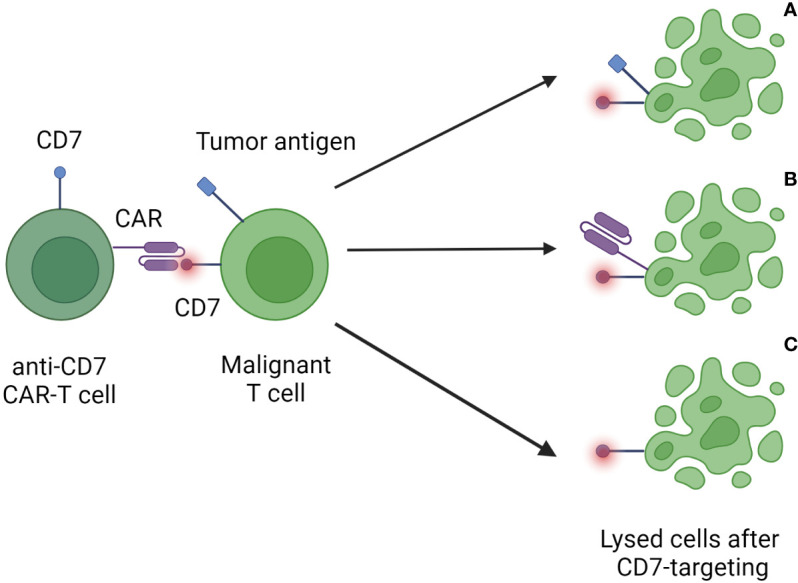
Fratricide elicited by anti-CD7 CAR-T-cell therapy. After infusion CAR-T cells recognize CD7 present on tumor cells **(A)**, other CAR-T cells **(B)**, and healthy T cells **(C)** in the recipient. These targeted cells are then destroyed by the immune system. The consequences are the depletion of the patient’s healthy T-cell reservoir as well as a reduced capacity and longevity of applied CAR-T cells.

An important issue in the use of anti-CD7 CAR-T cells against T-cell malignancies is the concept of “fratricide”, meaning the killing of therapeutic CAR-T cells by the CAR-T cells themselves due to shared expression of CD7 (**Figure 1**) (31). This protein is also expressed on NK cells, where it is related to activation and maturation (32). Fratricide results in reduced anti-tumoral activity, decreased survival of CAR-T cells and limited therapeutic success (33). Approaches to reduce fratricide include nanobody-based techniques (34, 35), natural selection of fratricide-resistant CAR-T cells (36, 37), and the use of antibodies (38) or protein expression blockers (39).

Chiesa et al. have recently shown the benefits of using base-editing to inactivate the genes encoding for the CD52 and CD7 receptors, and the β chain of the αβ TCR in a phase I trial of CD7-targeting CAR-T cells (40).The implementation of targeted pharmacotherapy with the objective of diminishing fratricide has the potential to facilitate the utilization of unedited anti-CD7 CAR-T cells, thereby enhancing the targetability of CD7. Previous studies have investigated the combination of ibrutinib and dasatinib for this purpose (41).

Another approach for the circumvention of fratricide is to make use of the naturally occurring subset of CD7^-^ T cells for the generation of CAR-T cells. These could be resistant to fratricide and provide a more sustained anti-tumor activity (42). Furthermore, to avoid fratricide, CD7 can be gene-edited with techniques like CRISPR/Cas9 (43, 44). If the expression of targeted antigens can be sufficiently reduced on the CAR-T cell itself, even bi-specific CAR-T cells are potential therapeutic tools without eliciting fratricide (45). These bi-specific CAR-T cells can be generated through the use of nanobodies as demonstrated by Xia et al. with the manufacturing of CAR-T cells targeting CD30 and CD5 (46).

Investigating another way to tackle fratricide, Jiang et al. have recently developed a “2-in-1 strategy” of knocking out the CD7 locus and inserting an EF1α-driven CD7-CAR in this locus to achieve improved tumor rejection in a mouse xenograft model (47). A similar technique was employed by Liao et al. for the generation of CD38-specific CAR-T cells (48). Similar to this is the editing of the T-cell receptor α constant (TRAC) locus (49). By this modification, allogeneic cell attack by T and NK cells can be reduced. There are multiple techniques to disrupt the TRAC expression, focusing on different parts of the receptor. The main technologies used for these editing approaches include CRISPR/Cas9 and TALENs (50). Xie et al. have recently demonstrated how CRISPR-based CD7- and TRAC-knockout CAR-T cells can efficiently proliferate and kill T-ALL cells *in vitro* and *in vivo*. The researchers showed increased frequency of CD8^-^ T cells and a higher number of activated CD4^+^ memory T cells (51). The application of gene-edited CD7^-^ hematopoietic stem cells could help sustain a sufficient number of healthy T cells after anti-CD7 CAR-T-cell therapy (52).

So far, clinical trials have utilized both autologous and allogeneic anti-CD7 CAR-T cells and have shown great promise regarding survival, tumor regression, and PFS (53–55). Recently, Chen et al. demonstrated the complete eradication of CD7^+^ T cells and the rapid expansion of the functional CD7^-^ T-cell subset following anti-CD7 CAR-T-cell therapy (56). Li et al. observed a patient achieving >3 years of PFS in their cohort of 12 patients treated with “off-the-shelf” anti-CD7 CAR-T-cell therapy (57). However, these studies have also confirmed the risks of T-cell aplasia as the result of on-target-off-tumor effects with associated severe adverse events like viral reactivation and infection, and fungal pneumonia. This is described by a case report of a hepatosplenic γδ-T-cell lymphoma treated with HLA fully-mismatched allogeneic anti-CD7 CAR-T-cell therapy. Researchers observed CRS, cytopenia and infections to a manageable extent and rapid decrease of circulating CAR-T cells after infusion. However, the patient did receive allo-HSCT and achieved lasting CR (58).

### 2.3 Potential targets

To circumvent CD7-related fratricide, researchers are also focusing on finding more suitable biological markers to differentiate between malignant, healthy, and therapeutic T cells. Recent studies have investigated the T-cell receptor β-chain constant domains TRBC1 and TRBC2 (24, 59), CD1a (60), CD2 (61), CD4 (62), CD5 (63, 64), CD21 (65), CD26 (66), CD38 (67), CD99 (68, 69), CCR9 (70), the natural CD7 ligand SECTM-1 (71), and the dual targeting of CD38 and LMP1 (72). Shaw et al. have shown the efficacy and antigen-specificity of TCRvβ-targeting CAR-T cells in cell lines, patient samples, and mice (73). To enhance the functionality of CAR-T-cells in the treatment of T-cell malignancies, researchers are also investigating other parts of the CAR-T-construct. This includes the hinge region, which has recently been shown to influence the cytotoxicity of anti-CD5 CAR-T cells and can be enhanced through specific modifications (74).

There are particular target antigens for different patient populations like targets specifically investigated for pediatric T-cell malignancies (75). Today, many of these targets seem promising, but CD7 is still the most extensively tested CAR-T-cell target antigen in clinical trials.

### 2.4 Increasing CAR-T-cell therapy safety

To reduce CAR-T-cell toxicities and adverse events, CAR-T cells incorporating “safety switches” or suicide genes have been proposed. These mechanisms limit the CAR-T-cell life span and persistence due to depletion upon administration of a prodrug (metabolic switch) or using monoclonal antibodies (76). In an effort to control CRS after CAR-T-cell therapy, Li et al. have shown promising results utilizing the Januskinase inhibitor ruxolitinib after infusion of anti-CD7 CAR-T cells (77). This application has also illustrated potential in preventing severe cases of CRS by limiting cytokine release and proliferation of CAR-T cells and is used for the treatment of CRS in other diseases (78). These advances towards a safer CAR-T-cell therapy are accompanied by innovations reducing the cost and complexity. These include retroviral vector-based gene therapy approaches based on *in vivo* delivery of the CAR gene (79).

To account for the risks and complexities of acquiring autologous T cells from patients, researchers are also investigating the application of allogeneic CAR-T cells. Hu et al. have shown the potential of allogeneic anti-CD7 CAR-T cells derived from healthy donors to target T-ALL cells (80). In the phase I trial, adverse events like high-grade ICANS and CRS or Graft-versus-Host-Disease (GvHD) were not observed in the study cohort of 11 patients. In contrast, Pan et al. administered allogeneic anti-CD7 CAR-T cells in 20 patients with 2 of them experiencing CRS grade 3-4 and 60% of patients experiencing GvHD grade 1 or 2 (53).

### 2.5 Cutaneous T-cell lymphoma

A special entity within the group of hematologic T-cell malignancies is the cutaneous T-cell lymphoma (CTCL), derived from the CD4^+^ T-cell subset. The most common forms of CTCL are mycosis fungoides and Sézary syndrome. While both diseases can be managed through treatment, achieving a complete cure is only feasible with allo-HSCT (81–83).

Currently investigated targets to combat CTCL with CAR-T cells include CD30, CD38, CD56 (84), and CCR4 (85). The latter is of particular interest as CCR4 is needed for cell trafficking and homing, as well as recruiting of regulatory T cells to the TME and has also shown to be upregulated in advanced stages of CTCL (86). However, anti-CCR4 CAR-T cells have been shown to elicit fratricide and selectively attack T_H2_-, T_H17_- and T_reg_-cells (87). Recent findings have indicated CD37 and TRBC as potential targets (88, 89). Gluud et al. have highlighted the importance of the JAK/STAT signaling pathway in CTCL and its potential targeting in immunotherapy (90).

Relevant obstacles in CAR-T-cell therapy for CTCL include antigen overlap with healthy T cells, transduction of malignant cells, fratricide, and prolonged T-cell aplasia (91). Tumor heterogeneity plays an important role in CTCL immunotherapy and shifts researcher’s attention towards dual- or multi-targeting therapy approaches. These could target malignant cells comprehensively and specifically (92). Further approaches include in-depth genotyping of tumor cells and monitoring of intra-tumoral activity of T cells (93). Due to these advances, CAR-T-cell therapy is seen as an option to achieve long-term remission in CTCL patients (94) and could offer a promising chance for these currently incurable diseases (95).

### 2.6 Peripheral T-cell lymphoma

These peripheral T-cell lymphomas (PTCL) are difficult to treat as there are few established treatment methods and the prognosis is mostly poor (96). Thus, PTCL proves to be a potentially rewarding target of CAR-T-cell therapy.

As shown in other T-cell malignancies, major problems include fratricide, the consequences of therapy-induced T-cell aplasia and the contamination of the CAR-T-cell product with malignant T cells (97). Similar problems in the implementation of CAR-T-cell therapy have also been independently described for entities such as T-follicular helper cell lymphoma (98).

Wu et al. have shown the efficacy of anti-CD30 CAR-T cells against PTCL cells *in vitro* and *in vivo* mouse models (99). As TRBC1/2 is already being investigated as a CAR-T-cell target antigen (24), current research is aiming at increasing the specificity towards this target in T-cell malignancies (100). A key aspect of this approach is the finding that T-cell malignancies are restricted to either TRBC1 or TRBC2. These can be targeted differentially. While clinical trials currently investigate other targets like CD5 and CD7 for T-cell malignancies, many of these trials do not include patients with PTCL (101). Other groups are investigating CD37, CD70, and CD147 as potential targets for PTCL CAR-T-cell therapy (102). As CD4 is also frequently upregulated on subtypes of PTCL like angioimmunoblastic T-cell lymphoma, anti-CD4 CAR-T cells are another area of investigation (103). Fang et al. could recently demonstrate the effect of CD4^-^/CD8^-^ anti-CD4 CAR-T cells against T-ALL and PTCL *in vitro* and *in vivo* without eliciting fratricide (104).

### 2.7 Outlook

CAR-T-cell therapy can offer a novel therapy option for patients with advanced, relapsed or therapy-refractory T-cell malignancies (105). While side effects could potentially be detrimental, the general risk-benefit evaluation could yield an overall positive outcome for a significant subgroup of patients. In a follow-up of 2 years after application of anti-CD7 CAR-T cells, Tan et al. observed durable efficacy but serious adverse events and potential disease relapse (106). Li et al. have recently highlighted the potential role of anti-CD7 CAR-T cells for bridging patients towards allo-HSCT (107).

In conclusion, anti-CD7 CAR-T-cell therapy remains the most promising approach to T-cell malignancies. So far, clinical trials with this target have shown potential regarding PFS and OS. However, other targets such as TRBC1/2, CD30, and specific targets for subgroups like CTCL and PTCL could provide potential benefits for patients.

## 3 Acute myeloid leukemia

### 3.1 Potential targets

Acute myeloid leukemia (AML) comprises a group of acute hematologic malignancies that arise from myeloid precursor cells and is often associated with a variety of genetic aberrations. As AML is the second most common type of leukemia in adults, there are numerous approaches to make this type of disease feasible to CAR-T-cell therapy (108, 109). Previous studies using CAR-T-cell therapy in AML patients have shown that the technique could prove a potentially valid strategy for patients with relapsed or refractory disease (110–114), a patient group that currently has only very limited therapy options.

Potential target antigens for CAR-T cells in AML include CD7 (110, 115), CD33/Siglec-3 (116, 117), CD38 (67, 111), CD41 (118), CD44 (119), CD64 (120), CD70 (121–123), CD117 (124), CD123 (125), CLL-1 (112, 114, 126, 127), B7-H3 (128), PR1 (129), FLT3 (130–132), IL1-RAP (133, 134), Siglec-6 (135), NKG2D (136), PRAME (137), and GRP78 (138). As it has recently been shown to be associated with the highly aggressive subcategory AML-MR, CD5 is another potential target of CAR-T cells (139). Similarly, CD36 is being investigated as a central driver of dissemination, disease progression, and relapse in AML patients and association with an unfavorable disease prognosis (140). In a high-precision approach, Giannakopoulou et al. have recently shown the possibility of targeting a single driver mutation (D835Y) in FLT3. This lead to the successful elimination of both CD34^+^ and CD34^-^ AML cells in mice through a mutation-specific TCR (141).

Other studies investigate bi-specific or dual CAR-T cells targeting CD13/TIM3 (142), CD123/FR-β (143), IL3-zetakine/CD33 (144), CD123/NKG2DLs (145), and FLT3scFv/NKG2D (113). These multi-targeted CAR-T-cell therapies could help to increase the specificity of CAR-T cells towards leukemic cells, limiting on-target-off-tumor effects and thereby enhance safety and efficacy for patients (146). Targeting multiple targets, especially those with low surface expression, can control tumor growth even in genetically heterogenous AML cases (147). Alberti et al. have recently proposed the dual targeting of stromal and non-stromal targets by CAR-T cells, investigating CD33/CD146 cytokine-induced killer cells (CIKs) (148). However, stromal syntenin appears to be downregulated in AML, potentially enhancing AML cell survival and increasing translational activity (149).

Kim et al. have also shown that the genetic inactivation of potential CAR-T-cell targets like CD33 on healthy hematopoietic stem cells might reduce the risk of bone marrow suppression (150). Studies investigating the role of CD33 have found this protein to be a non-vital marker of myeloid cells and its depletion does not hamper development and function of cells (151). Further studies underlined the role of CD33 on malignant AML cells by correlating its presence to clinically unfavorable outcomes and parameters (152). The presence of AML fusion-genes in CD33^-^ cells might be the reason for relapses after CD33-targeted AML therapy and necessitates the highly-precise engineering of CARs targeting this structure (153). This need is being addressed in current clinical trials through systematic preclinical structural evaluations (154). These evaluations also consider known modes of resistance to CD33-targeted therapy, including CD33-gene polymorphisms and upregulation of downstream pathways (155).

In another promising approach, Hino et al. have investigated the complex crosstalk between CAR-T cells and thymoid tissue in AML. The authors hypothesized a potential enhancement of patient’s endogenous anti-tumor capacities through elimination of tumor-antigen carrying APCs. Additionally, the thymus plays a central role in the development of the T-cell repertoire and could therefore be integral to the TME and success of immunotherapies for AML (156). Future research could investigate if the thymus is another possible focus of action to enhance the functionality of CAR-T-cell therapies.

The search for leukemia-specific target antigens remains a central challenge in the efforts to design CAR-T cells for AML therapy. While some targets are chosen because of their functional relevance to AML pathogenesis (CD5, CD33, CD36), others are of particular interest due to their relatively upregulated expression on AML cells or potency in pre-clinical trials (CD44, CD64, CD123).

### 3.2 Overcoming immunosuppression in AML

**Figure 2:**
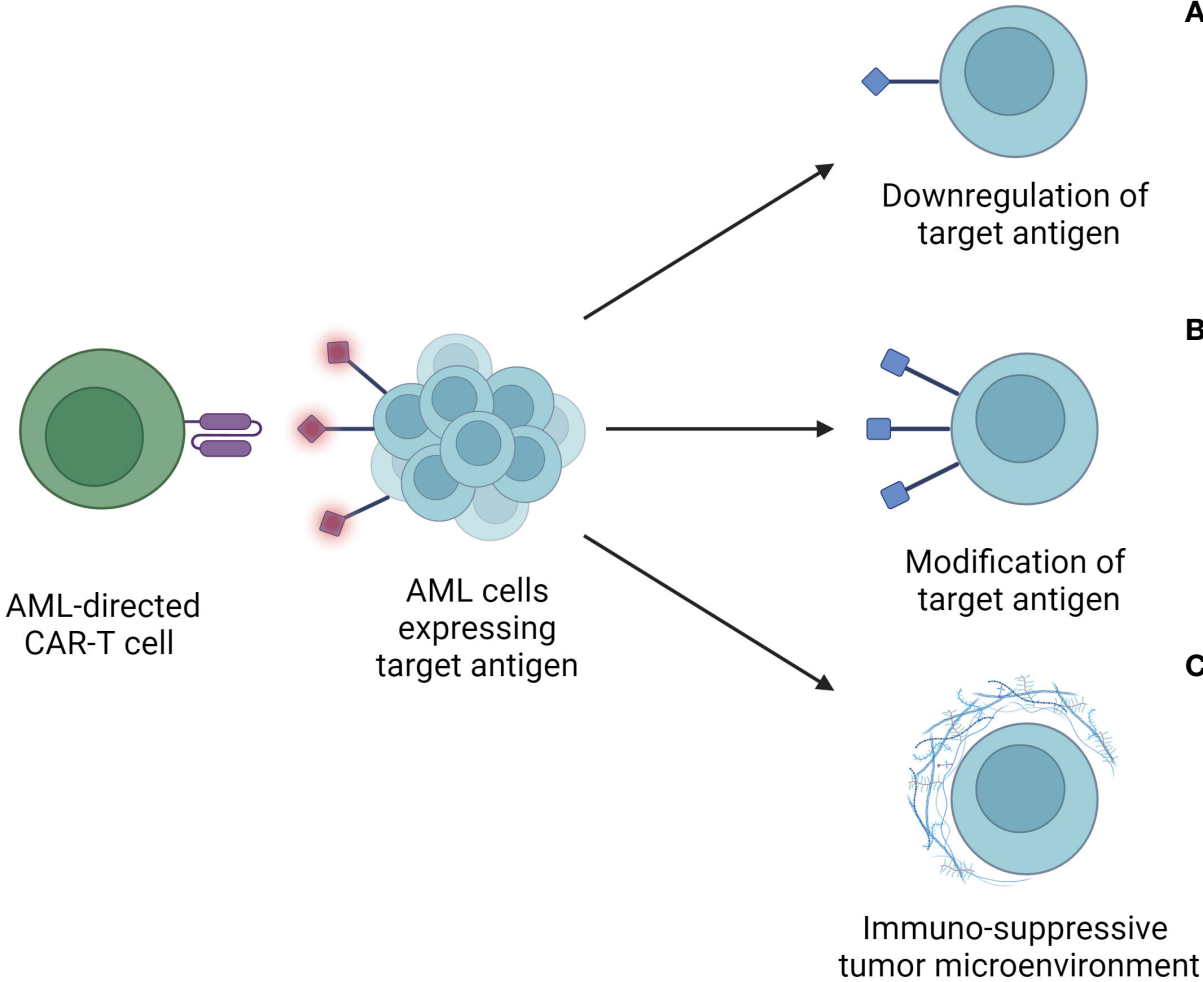
Escape mechanisms of AML cells after CAR-T-cell therapy. These include downregulation or loss of target antigen expression on tumor cells **(A)**, modification of the target antigen to escape recognition and binding by the CAR-T cell **(B)**, and an immunosuppressive tumor microenvironment (TME) **(C)**. Through these mechanisms, AML cells avoid detection and lysis through CAR-T cells, thus limiting the therapeutic efficacy of CAR-T cells.

To transfer the success of currently available CAR-T-cell therapies, researchers are focusing on a diverse range of approaches to enhance the immune response towards AML cells. Major obstacles towards this goal are summarized in **Figure 2**.

One of the most prominent challenges is the limited persistence of CAR-T cells ***in vivo*** and decreasing anti-tumoral effects over time with resulting disease relapse (157). Relapse can occur either with antigen-positive AML cells or accompanied by the phenomenon of antigen-loss. An important mechanism leading to antigen-loss is the selection of antigen-negative malignant clones through homozygous mutations of the B-cell receptor complex, splicing or target mutations. Newly developed, highly affine CAR-T cells have the ability to target even low antigen-expressing cells (158) and overcome epitope masking. The application of these T cells could help to target AML cells in patients after allo-HSCT with relapsed disease due to immune escape (159).

To further enhance CAR-T-cell efficacy in AML, An et al. and Leick et al. have shown positive effects of other fine-tuning approaches of CAR-T cells like the targeted down-regulation of PI3K-δ (160) and the design of a non-cleavable hinge region (161). Current studies also underline the potential use of unconventional T cells like invariant natural killer T cells and γδ-T cells, since they can function without HLA-signaling and possess natural anti-tumoral activity (162).

Naturally occurring mutations in target regions and molecular resistance mechanisms as well as an immuno-suppressive, leukemia-specific TME are further obstacles in effective CAR-T-cell therapy for AML (119, 163). As CAR-T-cell therapy is currently mostly investigated as a salvage therapy in relapsed or refractory disease, decreased T-cell fitness could play a role in the performance of CAR-T cells. In accordance with this, Vadakekolathu et al. have recently highlighted T-cell exhaustion and senescence as well as unique cellular signaling processes and chemokines as central components of this immunosuppressive microenvironment (164). This is supported by recent investigations into the role of ferroptosis signatures that highlighted their role in the TME and T-cell function (165). The effects of a functional T-cell immune response in AML patients on therapy efficacy (166), as well as clinical (167) and laboratory parameters (168, 169) are known.

### 3.3 Approaches to increase CAR-T specificity

Another obstacle of adoptive cell therapy in AML is the insufficient discrimination between healthy and malignant myeloid cells through specific antigens. This can lead to severe neutropenia and life-threatening infections due to myelosuppression (170). To overcome this, innovative techniques aim to increase the CAR-T-cell specificity in targeting malignant myeloid cells and therefore reduce on-target-off-tumor effects. Furthermore, researchers aim to increase the chance of long-term persistence of immunological tumor control.

To achieve these goals, researchers are employing multiple methods. These include suicide switches (anti-CD20 agents against CAR-T cells carrying CD20), logic gating (typically AND, NOT, and OR) to improve target recognition, and the genetic engineering of hematopoietic stem cells (HSCs) to reduce target antigen expression on their surface (171). Specific logic gating strategies have been proposed for anti-CD93 CAR-T cells (172) and an IF-BETTER gate for the targeting of ADGRE2 and CLEC12A (173).

So far, progress has been made in epitope-engineering with the altering of CD123 expressed on HSCs while maintaining their functional capabilities (174). Similarly, Casirati et al. have shown success in engineering epitopes of FLT3, CD123 and KIT and introducing these into CD34^+^ stem and progenitor cells resulting in the maintenance of functional hematopoiesis and the eradication of AML cells (175).. Another approach to sustain hematopoiesis is the targeting of leukemia-specific signaling pathways like the newly discovered IL33-ST2 interaction on myeloid leukemic stem cells (176). Nelde et al. have recently investigated non-mutated and neo-epitopes of common driver mutations of NPM1 and IDH2 shared between AML cells and leukemic progenitor and stem cells. This study further emphasizes the possible functional relevance of these epitopes and their association with clinical outcomes (177).

### 3.4 Novel CAR-T platforms

From a clinical perspective, the management of adverse events and therapy-associated risks is possibly more difficult due to the high number of older AML patients. This could intensify several adverse events including CRS, off-target-effects, and the risks associated with myelosuppression such as life-threatening infections (178).

To mitigate these risks, “suicide genes” have been implemented into CAR-T cells. This approach aims to control the infused suicide gene-modified CAR-T cells ***in vivo*** through its activation with a nontherapeutic agent like rapamycin (179). Constructs like this can lead to accelerated recovery of the patient’s immune system after CAR-T-cell therapy as demonstrated for anti-FLT3 CAR-T cells (180).

Wemke et al. have reported on first-in-human proof-of-concept for rapidly switchable anti-CD123 CAR-T cells based on the universal chimeric antigen receptor platform (UniCAR) that is currently further investigated in a phase Ia trial. This platform could allow for the sequential or parallel targeting of multiple target antigens. So far, all three patients who have gone through the treatment protocol in this study have experienced partial or complete remission without dose-limiting toxicities (125). CAR-constructs based on the UniCAR platform can be rapidly switched off upon the occurrence of adverse events by withholding the antigen targeting module. Ehninger et al. have shown the success of this approach for the handling of ICANS after administration of anti-CD123 CAR-T-cell therapy (181). These results are further supported by the reports of Peschke et al. who have developed an anti-FLT3 UniCAR-T-cell product. They observed killing of AML cells *in vitro* and *in vivo* in a murine xenograft model (182).

Nixdorf et al. have recently investigated the approach of using the AdCAR platform to specifically target AML cells using three adapter molecules targeting different antigens (CD33, CD123 and CLL-1). In these *ex vivo* experiments, AdCAR-T cells could be applied, targeted at one antigen, and later on re-targeted towards another, achieving high anti-tumor activity (183). This approach could address the problem of therapy-induced escape variants and target downregulation on AML cells. Using the UniCAR or AdCAR platforms, it could be possible to improve the management of CAR-T-cell induced adverse events like CRS and ICANS by limiting the exposure of CAR-T cells to their target antigen.

Overall, engineering CAR-T cells with mechanisms to reduce their activity after infusion appears as a central innovation towards making them a safer and more reliable therapeutic option. Novel CAR-T platforms provide the basis for innovative approaches, multi-targeting, and can help to address escape mechanisms and adverse clinical events.

### 3.5 Combinatorial regimens

Another field of investigation is the combination of CAR-T cells with established therapeutics like rapamycin (184), DNA hypomethylating agents like azacytidine (185) or decitabine (186, 187), and anti-PD-1 antibody therapy (166). Combinations of novel CAR-T-cell products with agents like chidamide and decitabine have proven superior to mono-therapy *in vitro* (122).

Using CAR-T cells to “bridge” the time towards allo-HSCT or in patients ineligible for allo-HSCT could help enhancing their usability in AML patients (188, 189). Anti-CD83 CAR-T cells even have the potential to be used in GvHD or AML relapse after allo-HSCT (190). Similarly, the combination with other pharmaceuticals might prove to be another therapeutic pathway (191, 192). However, major concerns against using CAR-T cells as a salvage therapy in AML relapse after HSCT include the occurrence of cytopenia and the risk of GvHD as well as limited efficacy (193). This may be due to increased T-cell exhaustion phenotype and a depletion in absolute T-cell numbers as hypothesized by Jia et al. using a newly developed Graft-versus-Leukemia (GvL) model in AML. The authors highlight the possibility of enhancing T-cell function in this model through the blockage of T-cell-inhibitory pathways (194).

As has been shown, valproic acid has the capacity to increase the cytotoxicity of anti-CD123 and anti-CLL-1 CAR-T cells against AML cells in mice (195). Cummins and Gill have recently highlighted the proposed advantages of either increasing antigen expression through combinatorial treatment or investigation of possible dose escalation schemes of the CAR-T-cell therapy (196).

### 3.6 Outlook

Despite disappointing progress in the field of immunotherapeutic approaches in AML (197), the implementation of proteomics (198) and transcriptomics (199) in designing a more personalized CAR-T-cell therapies holds promises. Single-cell sequencing offers the possibility to investigate mutational changes across the DNA, epigenetic and protein level (200). A recent review by Shahzad et al. has investigated 10 clinical trials and 3 case reports, overall calculating the response rate of patients with relapsed or refractory AML to CAR-T-cell therapy at 49.5% (201).

Finally, novel production pipelines of CAR-T cells like *piggyBac*-transposon-based technologies offer the possibility of cost reduction and shortening of production cycles (202). Approaches combining such transposon-based delivery systems with CRISPR technologies have the potential to facilitate allogeneic CAR-T-cell therapy through depletion of HLA-I and the TCR on donor cells (203). It should be noted that the induction of mutations and limited specificity restrict the utility of gene editing technology (204).

## 4 Chronic myeloid leukemia

### 4.1 Current state of treatment

Chronic myeloid leukemia (CML) is a disease with malignant transformation of myeloid precursor cells similar to AML. Unlike in AML, targeted therapies have significantly improved the treatment outcomes for CML using inhibitors of tyrosine kinases (TKI) – enzymes that are pathologically activated in most of the CML cases (205). Many patients can be treated safely and effectively with these oral inhibitors. Severe cases with progression into the acceleration phase or a potentially lethal blast crisis are relatively rare. Under optimal therapy and comprehensive management of comorbidities, patients can achieve a close to normal life expectancy (206).

However, there are patients that cannot be treated with TKIs, experience insufficient therapy success or excessive side effects (207). The current ultima ratio for CML patients is an allo-HSCT, which makes use of the highly advantageous GvL effect. Adoptive cell therapies could be employed as a salvage option for patients with relapsing disease even after allo-HSCT (208, 209) or long-time remission after TKI therapy (210).

### 4.2 Potential targets

In the past years, CD19 (211, 212), CD26 (213), CD38 (214), and IL-1-Receptor-associated Protein (IL-1-RAP) (215) have been investigated as potential target antigens for a CAR-T-cell therapy approach in CML. IL-1-RAP is of pronounced interest as its expression correlates with the formation of the Philadelphia chromosome in CML cells (216) and is often expressed in increased levels on malignant hematopoietic cells (217). CAR-T cells targeting IL-1-RAP can be produced semi-automatically and GMP-compliant since 2023 (218). In 2021, a digital droplet PCR (ddPCR)-based method was established to monitor both anti-CD19 and anti-IL-1-RAP CAR-T cells (219) to accurately assess their presence *in vivo*.

### 4.3 Outlook

Zhang et al. have shown that the combination of anti-CD19 CAR-T-cell therapy with the TKI dasatinib can potentially provide a curative therapy option even in the phenomenon of multilinear disease with several lines of mutated cells present (220). While several studies have shown substantial success in middle- to long-term follow-up of treated patients, the current research landscape on CAR-T cells focuses on other malignancies due to the success of TKI therapy (221).

Future studies have the potential to establish CAR-T-cell therapy as a valid and even potentially curative treatment option for CML through the targeting of promising antigens like IL-1-RAP. Especially patients refractory towards TKI therapy or after progression to acceleration phase/blast crisis can profit from this novel approach (222). Importantly, Jiang et al. have recently shown the preserved functional capacity of CD4^+^ T cells in CML patients, suggesting that this subgroup could be suitable for the generation of CAR-T cells (223). Additionally, success in implementing novel technologies like streamlined production processes and digital PCR allow for an easier implementation of CAR-T cells into clinical practice.

## 5 Chronic lymphocytic leukemia

### 5.1 Current state of treatment

Chronic lymphocytic leukemia (CLL) is a disease that primarily affects patients over the age of 65 years and is the most common type of Non-Hodgkin Lymphoma (NHL) in the western hemisphere (224). Today, there are numerous treatment options available for patients including monoclonal antibodies like rituximab and obinutuzumuab (anti-CD20), the BCL-2 inhibitor venetoclax, and BTK inhibitors (i.e. ibrutinib, acalabrutinib). Due to these available and well-established therapeutics with very high efficacy, research in CAR-T-cell therapy in CLL lacks behind that in other hematological malignancies of comparable clinical relevance (225).

Still, many patients develop a resistance towards established therapeutics (226). Disease progression into a highly malignant NHL (“Richter transformation”) remains a major issue (227) despite significant progress in the understanding of its genetics and mechanisms (228). Early results of phase I/II trials indicate a possible role for CAR-T cells in prevention or treatment of Richter transformation (229, 230), but highlight the importance of further research in this approach (231). Research has further identified IL-10, IL-6 and reduced levels of CD27^+^/CD45RO^-^/CD8^+^ T cells as potential biomarkers for refractoriness to immunotherapy (232). In contrast, IL-27 has been shown to boost CD8^+^ T-cell anti-tumor activity against CLL and is decreased in the peripheral blood of CLL patients (233). MALAT1 expression has recently been found to be associated with an aggressive course of disease in CLL and may hint towards previously not understood mechanisms of disease (234).

### 5.2 Overcoming resistance mechanisms

A challenging aspect of immunotherapy in CLL is a pathologically reduced immunocompetence. Main reasons for this include an immuno-suppressive microenvironment generated by malignant cells and systemic extracellular vesicles (235), changes in immune-synaptic signaling (236), and disruptions in T-cell metabolism (237). Additionally, T_reg_ cells seem to disturb the expression of CD62L and IL-4R on neutrophils, reducing their natural immunological capacities in CLL models (238).

T cells of CLL patients frequently display an exhausted phenotype and show increased expression of PD-1, CTLA-4, TIGIT, CD160, and CD244 (239). Agarwal et al. have recently demonstrated how the selective deletion of CTLA4 can enable CD28 signaling in CAR-T cells targeting CD19 in CLL (240). Epigenetic studies have highlighted changes in the profile of cytokine secretion, reduced cytotoxic capacities, and exhaustion as factors leading to reduced T-cell function in CLL (241). Possible solutions include the application of allogeneic CAR-T cells that are not affected by these defects or the combination with modulators of epigenetic reading (242).

**Figure 3:**
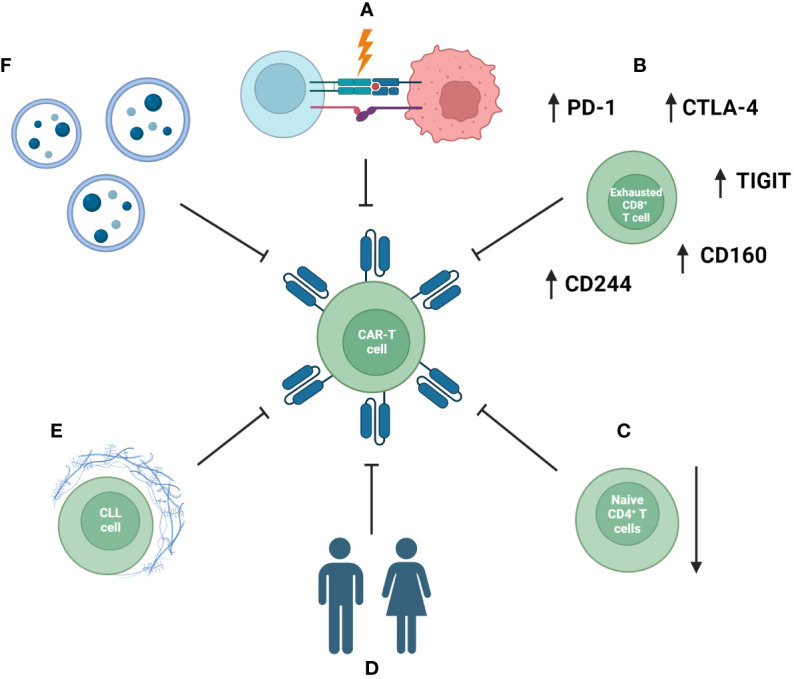
Mechanisms impacting the efficacy of CAR-T-cell therapy in CLL patients. These include disruption of formation of the immunological synapse and communication between an immune cell and a tumor cell **(A)**, exhausted phenotype of CAR-T cells **(B)** and a reduced naïve compartment of T cells **(C)**. Additionally, patient-specific factors and comorbidities **(D)**, an immunosuppressive tumor microenvironment **(E)**, as well as systemic extracellular vesicles (EVs) inducing exhaustion phenotype CAR-T cells with reduced anti-tumor efficacy **(F)** are important factors.

T-cell-focused studies have further indicated the CLL-associated depletion of polyfunctional CD26^+^ T cells, which represent another possible target for adoptive cell therapy (243) and unique subsets and transcriptional signatures of T cells in CLL patients (244). These pathological mechanisms can disrupt the efficacy of CAR-T cells as well as their long-term establishment in the recipient (**Figure 3**) (245).

Specific T cells like Vγ9Vδ2 cells have been identified as a central player in the TME and γδ-T cells can be used as carriers of anti-CD19 CARs (246–248). Investigations into different ways of cultivating Vγ9Vδ2 cells and using them as cytotoxic agents are showing promising results (249). Engineered γδ-T cells have further been shown to impact the TME and potentially show lower rates of on-target-off-tumor effects (250). Donor-derived γδ-T cells could be another therapeutic option as they exhibit natural anti-tumor activity (251). Another aspect of fine-tuning γδ-T cells for antitumor use includes the specific expression of chemokines like IL-15 to increase longevity and tumor control (252).

Recently, receptors of the SLAM-family have been investigated as central players of disease progression. So far, SLAMF3 and SLAMF7 are in focus of CAR-T-cell research. However, these approaches have been limited by fratricide induced by SLAMF-targeting CAR-T cells (253).

### 5.3 Previous studies

CAR-T-cell therapy has been employed since 2011 for CLL patients (254). While the first CAR-T-cell treated CLL patients have shown both comprehensive as well as sustained responses (255, 256), this is not the case for all patients. Using a CD19-targeting CAR-T-cell therapy (lisocabtagene maraleucel, initially approved for DLBCL, PMBCL and FL), 45% of patients after multiple disease relapses achieved a complete remission (257). In 2020, Cappell et al. demonstrated a duration of response of over 3 years for 50% of CLL patients treated with anti-CD19 CAR-T-cell therapy with a limited prevalence of serious adverse events and a median event free survival of 40.5 months (258).

Recently, Siddiqi et al. have reported a phase I/II study investigating lisocabtagene maraleucel in 117 patients who had previously experienced BTK-inhibitor therapy failure. The investigators observed a complete response rate of 18% with 9% of patients experiencing grade 3 CRS and 18% experiencing grade 3 neurological events. Overall, within 90 days after CAR-T-cell infusion 5 treatment-emergent deaths were reported (259).

Detailed investigations into the kinetics of CAR-T-cell persistence have shown correlations between the numbers of CAR-T cells present in the peripheral blood and adverse events like the occurrence of CRS (260). In line with these findings, patients treated with anti-CD19 CAR-T-cell therapy for CLL have experienced side effects like bacterial, viral, and fungal infections due to myelosuppression similar to patients suffering from other hematological malignancies (261). Through the comprehensive analysis of 47 patients with CLL or Richter transformation receiving anti-CD19 CAR-T-cell therapy, Liang et al. have determined several factors associated with longer PFS. These include peak CD4^+^ and CD8^+^ CAR-T-cell expansion, MRD negativity and CAR-T-cell persistence (262).

Other studies underline the complex immune mechanisms influencing the efficacy, survival and functionality of CAR-T cells (263). Until today, CD19 is still the most comprehensively investigated target antigen for CAR-T-cell therapies directed against CLL (264). However, there have also been promising results with the use of CARs targeting malignancy-associated κ-light chains (265), CD32b (266), the Fc μ receptor (267), and Siglec-6 (268) in CLL patients. Luo, Qie et al. have recently reported the success of BAFF-R-targeted CAR-T cells even in CD19^-^ cell lines (269).

### 5.4 Outlook

Currently, CAR-T-cells are being investigated as part of an integrated therapeutic algorithm including established lines of therapy to maximize the percentage of patients being treated optimally (270). Studies have shown favorable results for the implementation of CAR-T cells into the therapeutic repertoire for CLL through combination with PI3K-γ-δ inhibitors (idelalisib) (271, 272), ibrutinib (273, 274), and lenalidomide (275). The sequential application of BTK inhibitors and CAR-T cells seems to provide synergistic effects in CLL treatment (276).

The combination of fludarabine and cyclophosphamide prior to anti-CD19 CAR-T-cell infusion has been shown to improve CAR-T-cell functionality and clinical outcomes, a phenomenon also seen in other hematological malignancies (277). Studies suggest favorable effects of these combinations on severe CAR-T-cell associated side effects like CRS (278). Several advantages set CAR-T-cell therapy apart from options like allo-HSCT: possibly milder profile of adverse events, shorter duration of treatment, and the prospect of long-term CAR-T-cell persistence and therefore disease control (279).

However, there is still a multitude of factors keeping CAR-T-cell therapy from becoming a widely available and reliably applicable therapy for CLL (280, 281). CAR-T-cell therapy could prove especially viable for patients suffering from refractory or relapsed disease, as well as those developing secondary malignancies or suffering from intense symptoms (282). In a subset of patients, CAR-T-cell therapy can offer a potential way to disease eradication (283). Additionally, recent approaches like the killing of CLL cells through targeting of the Lck-IP3R protein-protein interaction hint towards other innovative methods of immunotherapy targeting in CLL (284).

## 6 Classical Hodgkin-Lymphoma

### 6.1 Current state of treatment

Classical Hodgkin-Lymphoma (HL) is considered a highly curable disease today. The majority of patients achieve deep and long-lasting complete remission with standard therapy (285). However, cases of refractory or relapsing disease still pose an important problem (286). Furthermore, treatment-related toxicities and side effects, especially in individuals over the age of 60 years, still prove life-threatening and therapy-limiting.

### 6.2 CD30 and the TME in HL

CAR-T-cell investigations in HL patients mainly focus on CD30 as a target antigen (287) due to the abundant expression on malignant Hodgkin Reed-Sternberg cells and malignant B cells (288, 289). Previously, this expression has successfully been exploited through the antibody-drug conjugate (ADC) brentuximab–vedotin (BV) targeting CD30 (290). CD30 plays an important pathophysiological role in cell-morphology and chromosomal instability (291). First phase I and II trials utilizing anti-CD30 CAR-T cells showed substantial success in heavily pre-treated patients with an overall response rate of 39-72% and mostly tolerable side effects (292, 293).

Since these initial clinical trials, the RELY-30 study has shown further improvements in the safety profile and efficacy of anti-CD30 CAR-T-cell therapy but also limited durability of responses with 36% 1-year PFS and additional relapses occurring after this point of time (294). Kim et al. have also reported a decrease or loss of CD30 expression in relapse after targeting through different modes of immunotherapy (295).

Since recent research has highlighted the importance of the TME in HL, it has become a target of CAR-T-cell therapy approaches (296, 297). Studies have investigated CAR-T-cell-mediated targeting of CD19 (298) and CD123 (299) to influence the TME of HL cells and induce a long-lasting immune response to lymphoma cells. These substances could potentially be combined with anti-CD30 CAR-T cells to achieve synergistic effects (300). Such an effect has been shown *in vitro* for the combined targeting of CD30 and CCR4 (301). The varying persistence of CD30 expression on malignant cells could mean that antigenic escape is unlikely to occur in CAR-T-cell therapy (302). Like CD30, the expression and signaling of PD-1 and PD-L1 is highly characteristic for Hodgkin Reed-Sternberg cells and could prove a valuable target in HL patients (303).

### 6.3 Outlook

Since CD30 has already been shown to be a potentially targetable structure, CAR-T-cell research for HL focuses on optimization of lymphodepletion regimens (304), improvement and high-precision engineering of CAR-T cells (305, 306), and the development of combinatorial regimens (307) with agents like PD-1 inhibitors (308) or allo-HSCT (309). Thereby, CAR-T-cell therapy can provide a path for refractory patients after BV and checkpoint-inhibitor therapy who lack clear and defined therapy options in current guidelines (310). Recent results from the CHARIOT trial have shown a generally safe side effect profile for CAR-T-cell therapy in a cohort of heavily pretreated HL patients (311).

More clinical trials are needed, but anti-CD30 CAR-T-cell therapy could prove to be a new tool in the treatment arsenal in HL. It can provide an option for patients insufficiently responding to conventional therapy (312). Reduction of toxicities, optimization of CAR-design and combination regimens with other therapeutic agents are further areas of promising research (313). The integration of technologies like mass cytometry, single-cell RNA sequencing, and monitoring of circulating tumor DNA promise more detailed insights into the pathophysiology of HL and potential new molecular targets (314).

## 7 Burkitt-Lymphoma

### 7.1 Current state of treatment

Burkitt-Lymphoma (BL) is a highly aggressive NHL and commonly found in children and adolescents. BL cases are categorized accordingly to their etiology into endemic, sporadic, and immunodeficiency-associated (315). Long-term remission through high-intensity chemotherapy is achieved in over 90% of pediatric but only 75-85% of adult patients (316).

The application of intense chemotherapy regimens in adult patients is especially limited by the occurrence of toxic side-effects. Treatment failure among adult patients is common and occurs in up to 35% of patients (3-year PFS of 64%) (317). Thus, new and comprehensive treatment options for refractory and relapsed disease as well as for special patient cohorts are needed.

### 7.2 Current research

Case reports have shown considerable success in the treatment of relapsed and refractory BL cases (318–327), mostly utilizing CAR-T-cell therapies targeting CD19. These reports include both adult and pediatric BL cases and show substantial therapeutic responses in both patient groups. Despite this success, a recent case series by Geerts et al. has also shown a case of BL disease progression and patient death despite objective CAR-T-cell expansion *in vivo* and adequate management of side effects (CRS grade 2) (328). A recent case report has highlighted the possibility of using unedited HLA-matched allogeneic CAR-T-cells, in this case directed against CD20 and CD22, for the treatment of BL. Due to persistent pancytopenia, the patient also received an allo-HSCT from the same donor 55 days after application of the CAR-T-cell therapy (329).

**Figure 4:**
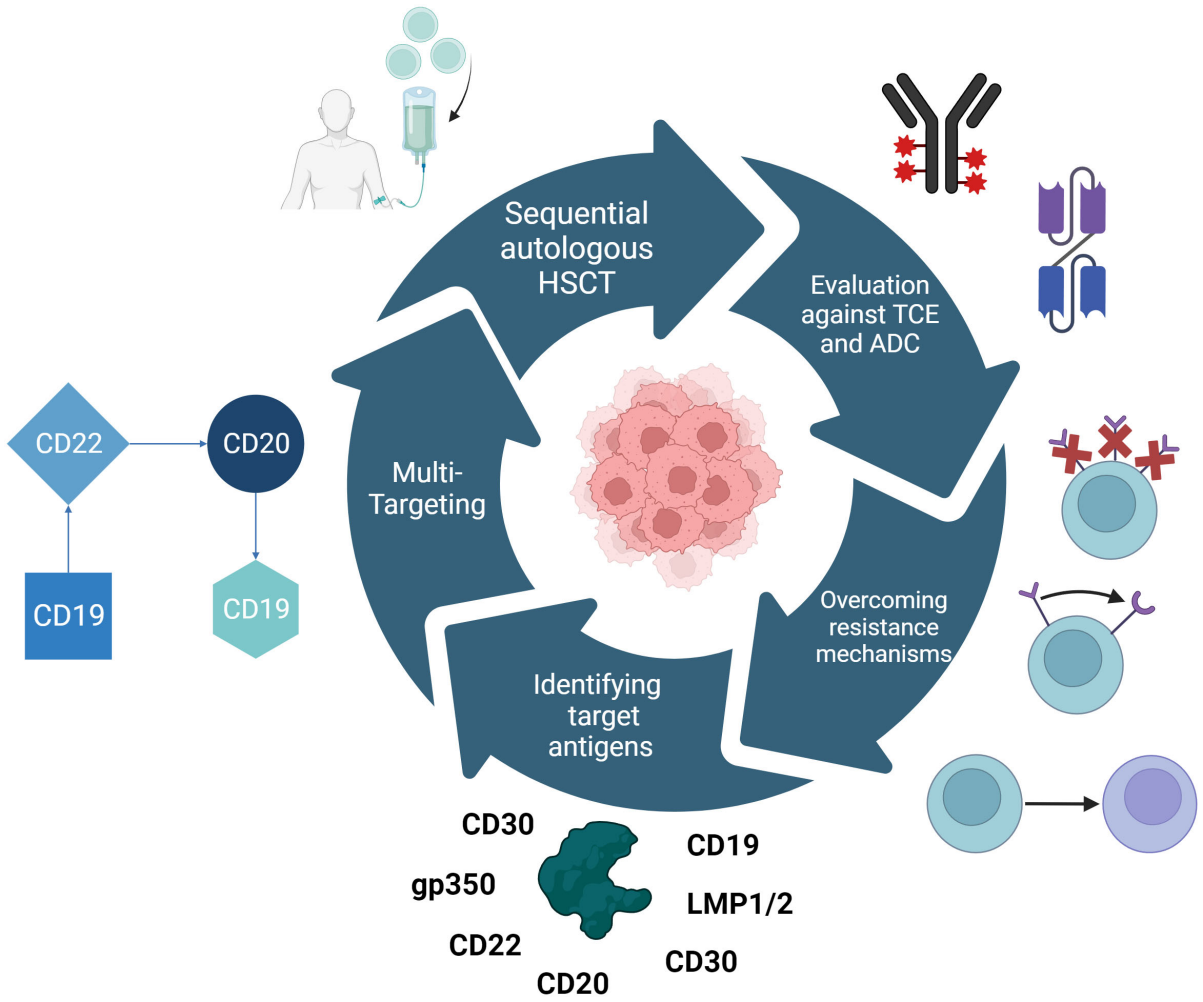
Research approaches for making CAR-T-cell therapy useable for Burkitt lymphoma as defined by the ACCELERATE study group. These are the identification of novel target antigens and the repurposing of those already in use for other hematologic malignancies, multi-targeting CAR therapy to prevent antigen escape as currently employed with CD19, CD20, and CD22. Furthermore, CAR-T-cell therapy needs to be evaluated in the context of combinational therapies e.g. sequential autologous HSCT, T-cell engagers (TCEs), and antibody-drug conjugates (ADCs). Challenging resistance mechanisms that need to be overcome include the downregulation of targeted antigens, structural changes in these antigens or the switch of myeloid linages.

Hsieh and Rouce have comprehensively compiled three major targets of research to make CAR-T-cell therapy usable in Burkitt-Lymphoma and other pediatric hematologic malignancies: Tackling the immunosuppressive TME, antigen escape mechanisms, and optimizing CAR-T-cell efficacy and functionality (330). This was further refined by the ACCELERATE study group to resistance mechanisms, best tumor targets, possibilities of double-/triple-targeting, and the evaluation of CAR-T-cell therapy in the context of T-cell engagers, ADCs, and autologous HSCT (**Figure 4**) (331).

To tackle these challenges, Laurent et al. have employed high-resolution investigations into the phenotype of post-CAR-T-cell therapy cancers and have shown a highly variable decrease of one or more B-cell markers in relapsed patients (332). These findings indicate extensive genetic changes, remodeling and acquired mutations in PI3K- and KRAS-pathways, leading to an impaired B-cell differentiation.

### 7.3 Combinatorial regimens

In line with these laboratory findings, Du et al. reported promising outcomes of a pediatric patient treated sequentially with anti-CD19, -CD22 and -CD20 CAR-T cells (319). This was expanded upon by Zhang et al. in a clinical trial with 5 pediatric patients each achieving complete remission through the sequential application of anti-CD19, anti-CD22 and anti-CD20 CAR-T cells, depending on histological tumor staining analyses (333). In the largest trial of its kind in 2022, Liu et al. treated 23 patients with up to 4 cycles of CAR-T-cell-therapy: 23 with anti-CD19, 13 of those with anti-CD22, 6 of those with anti-CD20, and 1 patient with a second anti-CD19 CAR-T-cell therapy (334). Over the course of the four therapy cycles, 18 of 23 patients achieved complete remission and 4 patients died due to rapid disease progression or CNS involvement with intracranial mass progression.

Further studies have investigated the combination of anti-CD19/CD22 CAR-T-cell therapy with allo-HSCT (335) and showed an overall survival rate of 55.6% in a cohort of heavily pretreated r/r BL patients. The investigators highlighted the beneficial effects of early initiation of CAR-T-cell therapy in combination with allo-HSCT. There is evidence that CAR-T-cell therapy could also play a valuable role in bridging BL patients towards allo-HSCT (336). Conversely, established therapeutic approaches like radiation therapy are also investigated as possible bridges towards CAR-T-cell therapy with the goal of reducing tumor load in high-risk patients (337).

Like in other hematological malignancies, the combination of established therapeutic agents with CAR-T-cell therapies is being investigated in BL patients. This includes the histone deacetylase inhibitor romidepsin that induced BL cell death *in vitro* and *in vivo* mouse models when combined with anti-CD20 CAR-T cells (338).

### 7.4 Outlook

Translational approaches in CAR-T-cell design also include targeting of EBV-associated structures like the abundantly found gp350, as EBV-infection is frequently associated with BL (339). *In vivo* studies in mouse models have so far shown limited success in reducing EBV DNA load, tumor development and growth, and inflammation parameters (340, 341). Further CAR-T-cell targets to combat EBV in lymphoma tissue include latent membrane proteins LMP1 and LMP2 (340), and Gb3 (342).

In conclusion, Burkitt-Lymphoma appears to be a promising target for CAR-T-cell therapy. Especially the availability of CD30 as a potential target, the possibility of employing sequential regimens of CAR-T cells targeting different B-cell markers and substantial response rates in initial clinical trials may provide hope for the successful treatment of patients with relapsed or refractory disease. Future research could offer the possibility of establishing CAR-T-cell therapy in the context of other established therapeutics, and larger and more diverse patient cohorts.

## 8 Hairy cell leukemia

### 8.1 Current state of treatment

Hairy cell leukemia (HCL) is an uncommon type of B-cell NHL. It rarely presents in a prolymphocytic, more aggressive variant known as HCL-v. Both diseases can be controlled through established therapy regimens including cladribine and pentostatine as first-line therapies. More than 95% of patients can be treated adequately with these therapies (343).

However, nearly 50% of all patients encounter disease relapse within 10 years, often being unable to undergo first-line treatment again due to novel mutations that induce resistance and the need for more intensive therapeutic approaches (344). Currently, proposed therapeutic options for r/r HCL include BRAF-inhibitors, BTK-inhibitors and immunotherapy targeting CD22 (345). Further, HCL-v patients are faced with inferior outcomes after standard therapy lines (346). HCL can also arise from Richter-transformation of CLL, a rare occurrence without defined therapeutic pathways (347). Therefore, these patient groups can potentially benefit from CAR-T-cell therapy (348).

### 8.2 Potential targets

A challenging aspect of CAR-T-cell therapy targeting HCL/-v is the diverse expression profile of surface antigens on malignant cells. While HCL cells usually express CD103, CD123, CD25, and CD11c abundantly, other markers that have been targeted in other CAR-T-cell trials like CD5, CD26 or CD38 are expressed very heterogeneously (349). This is an additional difficulty in differentiating HCL from other hematological malignancies and can be addressed by technologies like flow cytometry (CD5, CD200) (350, 351). Bhatti et al. have reported on 10 HCL cases with all of them expressing CD11c, CD19, CD20, CD22, and CD123 (352). HCL-v is often characterized by lower expression of CD25 and CD123 (353). Furthermore, there are reports of CD10^+/-^ biclonality (354), CD103^-^ cells (355), variable expression of CD123 on HCL-v cells (356), or cases with expression of both CD38 and CD10 (357).

Maitre et al. have recently approached this problem through a comprehensive gene expression analysis that could provide the basis for identification of novel therapeutic targets (358). Another potential target is ROR1 (359), already investigated for therapeutic use in patients with CLL, MCL, and ALL (360). Current research has also shown success in utilizing anti-CD22 immunotoxins, proving this could be another experimental target of HCL/HCL-v CAR-T-cell therapy (361).

### 8.3 Outlook

Overall, CD123 and more established CAR-T-cell targets like CD19/20/22 are the most promising targets for CAR-T-cell therapy in HCL (362, 363). The generally low and at best variable expression on the cells of the aggressive HCL-v is an important problem keeping CAR-T-cell therapy from being implemented for the most pressing cases of HCL. This can be addressed through comprehensive analysis of antigen expression and diagnostics, establishment of sequential therapy regimens and further research into novel HCL-specific target antigens. According to current knowledge, a full flow cytometric HCL/-v panel could include CD5, CD10, CD11c, CD19, CD20, CD22, CD25, CD26, CD38, CD103, CD123, and CD200 to comprehensively assess marker expression and individual therapy options.

## 9 Waldenström’s macroglobulinemia

### 9.1 Current state of treatment

Waldenström’s macroglobulinemia (WM), also called Lymphoplasmacytic Lymphoma, is an indolent NHL characterized by infiltration of multiple malignant lymphocytic cells (plasma cells, plasmacytoid lymphocytes, lymphocytes) into the bone marrow (364). These cells secrete monoclonal IgM and show constitutive B-cell activation signaling (365). WM is commonly treated with immune-chemotherapy or BTK-inhibitors. These therapy lines often lead to remission but either induce significant side effects (ICT) or necessitate constant therapy (BTK-inhibitors) (366).

Currently available therapies are not curative for WM and no consensus on the adequate treatment of relapsed or refractory disease exists. Main therapy regimens include toxicity- and progression-free-oriented application of differential therapy lines (367, 368). Ahmed et al. have shown how diverse factors like complex karyotype and refractoriness to multiple lines of therapy significantly impact prognosis after auto-HSCT. Patients receiving auto-HSCT still experience disease relapse in 46% of cases (369).

### 9.2 Potential targets

Palomba et al. have reported the treatment of three patients using anti-CD19 CAR-T-cell therapy and clinical remission in all patients but also disease recurrence 3 – 26 months after infusion (370). Another case report has shown more promising results in a patient suffering from histologically transformed disease after treatment with anti-CD19 CAR-T-cell therapy. The patient reached complete remission and remained in this state for 12 months up to the publication of the case (371).

Other investigated targets include NF-κB and MALT1. However, their usability for CAR-T-cell therapy has not yet been explored (372). CD40 is another promising target since it plays a central role in shaping the TME and has an impact on WM cell growth (373). Qiu et al. have recently proposed several distinct cell lines in WM patients with a rare entity of CD3^+^/CD19^+^ cells with “stemness” features (374). This cellular distinct and potentially crucial subgroup could be a central target to effectively combat WM since CD19 is an established target antigen of CAR-T-cell therapy in other diseases.

BCMA, the target antigen of currently approved CAR-T-cell therapies in Multiple Myeloma has also been shown to be elevated in the blood of WM patients (375). CD5 is expressed in up to 50% of WM cases and could be a CAR-T-cell target in these patients (376). Kaiser et al. have recently highlighted the role of CXCR4 in WM and its possible use as a therapeutic target (377). Additionally, CD138 has recently emerged as a potentially identifying feature of WM tumor cells, associated with IgM peaks and MYD88 mutations (378). Recent research has shown the role of nanoscale organization of CARs and TCRs for CAR-T cells targeting CD138 with yet unknown consequences (379). Future investigations can determine whether these structures are suitable targets for CAR-T-cell therapy in WM.

### 9.3 Outlook

Despite the limited research conducted so far in the field of CAR-T-cell therapy in WM, the success of treating CLL with anti-CD19 CAR-T cells sparked hope to translate these findings (380). This hope is supported by recent investigations, indicating that T-cell number, distribution, and functionality in WM patients are conserved, in contrast to CLL patients (381).

Still, other potentially WM-specific targets could prove to be even more suitable. Intra-tumor heterogeneity is of particular interest in WM as it increases the difficulty of defining targets sufficiently broadly expressed on tumor cells (364). Especially young patients, who might tolerate the potential side effects of a CAR-T-cell therapy better, could profit from the curative therapeutic approach (382).

## 10 Discussion

### 10.1 Common challenges and approaches

In this review, our objective was to outline the potential uses of CAR-T-cell therapy in emerging indications that extend far beyond the hematologic diseases currently approved for treatment. We have discussed the use of targets already established in different malignancies, the identification of novel targets, and innovative approaches of defining these.

In a recent review, Mishra et al. have highlighted antigen loss after CAR-T-cell therapy as a major driver of treatment failure and compiled several main reasons: Genetic alterations of antigens, epigenetic modifications (methylation), development of immunosuppressive escape mechanisms, clonal selection of antigen-negative subclones, and antigen shedding into the TME (383). Current research focuses on many of these mechanisms to enhance and sustain CAR-T-cell functionality *in vivo* (384).

While many of the diseases we have addressed are already approachable with currently available therapies, none of them provide comprehensive treatment options to refractory or relapsed disease status. CAR-T cells have the potential to fill this gap and provide a potential curative treatment option in many currently incurable hematologic malignancies (385).

### 10.2 Integration of CAR-T-cell therapy into clinical practice

While the potential side effects and costs of the technology still hinder its extensive implementation into clinical practice, these problems are being tackled and are expected to be resolved or at least substantially improved in the near future (386, 387). To make these advances available to patients and employ them for improvement of care, CAR-T-cell therapy needs to be economically feasible, inclusive, and access for all patient groups must be ensured (388–390). A recently proposed approach to these requirements is the “Cocoon Platform” by Lonza (Basel, Switzerland) (391). A distinct advantage of CAR-T-cell therapy over currently available treatment options such as tyrosine-kinase inhibitors is that patients may experience sustained long-term remissions after one single CAR-T-cell therapy and therefore are spared the burden of continuous treatment regimens (255).

Further research highlights the potential application of CAR-T-cell therapy in the context of combinatorial, bridging or sequential treatment modalities. These types of regimens have been implemented into clinical practice for a long time and promise a personalized and highly refined therapeutic approach for each individual patient. In comparison to this, several research approaches highlight the possibility of establishing multi- or “pan-leukemic” targets (392, 393). A promising candidate is CD45, which has recently been investigated in an epitope base editing approach. Through this modification, Wellhausen et al. were able to design cells with the ability to engraft, persist and differentiate in an ***in vivo*** model and were not attacked by anti-CD45 CAR-T cells. This effect could be shown for models of AML, B-cell lymphoma and T-ALL (394).

The recently developed YTB323 CAR-T-cell therapy, based on the anti-CD19 tisagenleucel, is another particularly promising approach. Through the novel T-Charge manufacturing platform, this therapy can be provided to patients within less than 10 days after leukapheresis (395). Potential benefits include an enhanced clinical safety profile, high response rates and preservation of a higher T-cell stemness. This final aspect is assumed to play a major role in CAR-T-cell functionality and survival (396).

Future research is urgently needed to expand upon our current knowledge of the applicability of CAR-T-cell therapy in other diseases and particular clinical courses. Clinical research needs to include currently underrepresented disease groups into CAR-T-cell trials to broadly establish them in the clinical practice (397).

### 10.3 The role of AI in CAR-T-cell research

Artificial intelligence (AI) and machine learning (ML) are two of the most fascinating topics in the currently evolving medical research landscape (398). Their effect on multiple disciplines cannot yet be reliably estimated but early reports hint towards their relevance in tackling biological and structural challenges (399). Because of an intense attention to the topic, hematologic research is also investigating potential uses of the technologies in the diagnosis and treatment of hematologic malignancies.

In fact, there are several areas of CAR-T-cell therapy in which AI and ML can benefit patients and clinicians. One key example might be the prediction and assessment of adverse events like CRS (400–402). Since early detection of an overbearing immune response is of critical importance to adequately support patients, this could prove beneficial to improve clinical outcome and therapy management. Similarly, AI models can assess clinical parameters for their prognostic value and predict long-term outcome (403). This includes the close investigation of the crosstalk between CAR-T cells and the human gut microbiome, a challenging task that requires the collection, curation and handling of large datasets (404).

Identification of possible targets for CAR-T-cell therapy represents another excellent challenge to be solved by AI. Through multi-OMIC approaches, researchers have a previously unknown amount of data at their disposal to investigate potential targets for CAR-T-cell therapy (405). ML algorithms can aid in filtering, clustering, and interpreting these data to assess neoantigens or design TCRs (406, 407). Through the implementation of multi-dimensional ML algorithms it is possible to investigate large datasets on CAR-T-cell phenotype (408) as well as correlations between cellular and clinical data (409).

As the production of CAR-T cells is highly elaborate and requires multiple complex steps as well as close monitoring and data collection for quality and process control, researchers are employing AI to determine optimal production conditions and workflows (410–412). Similar approaches have shown success in other biological production systems. Since the clinical performance of CAR-T-cell therapy is highly dependent on the quality of its production, previous promising results in improving cell culture and expansion can hopefully be translated to similar processes in CAR-T-cell technology (413).

Gil and Grajek have recently outlined potential applications of AI in CAR-T-cell therapy. These include: Improvement of lymphodepletion regimens, identification of novel target antigens, designing of new therapeutic molecules, and the construction of predictive clinical models based on biomarkers, antigen loss, TME and T-cell phenotype (414). Furthermore, AI can aid in the improvement of gene editing technologies employed for CAR-T-cell design, the combination of currently separately tested approaches (expression of cytokines or transcription factors), and the screening of large CAR-T-cell libraries (415).

In conclusion, AI can potentially address a number of the most pressing issues in the application of CAR-T-cell therapy for hematologic malignancies. Both clinical as well as experimental problems can potentially be approached through AI and ML, especially to harness large datasets, predict novel designs, and improve high-precision workflows. This decade of CAR-T-cell and AI research will show if the technology can live up to these expectations.

## Author contributions

HK: Conceptualization, Investigation, Methodology, Visualization, Writing – original draft, Writing – review & editing. LM: Methodology, Visualization, Writing – review & editing. SC: Investigation, Validation, Visualization, Writing – review & editing. WF: Supervision, Visualization, Writing – review & editing, Writing – original draft. WA: Supervision, Visualization, Writing – review & editing. AB: Conceptualization, Funding acquisition, Methodology, Project administration, Resources, Supervision, Validation, Visualization, Writing – original draft, Writing – review & editing.
